# Evaluation of Physical and Mechanical Properties of Porous Poly (Ethylene Glycol)-co-(L-Lactic Acid) Hydrogels during Degradation

**DOI:** 10.1371/journal.pone.0060728

**Published:** 2013-04-09

**Authors:** Yu-Chieh Chiu, Sevi Kocagöz, Jeffery C. Larson, Eric M. Brey

**Affiliations:** 1 Department of Biomedical Engineering, Illinois Institute of Technology, Chicago, Illinois, United States of America; 2 Research Service, Hines Veterans Administration Hospital, Hines, Illinois, United States of America; Instituto de Engenharia Biomédica, University of Porto, Portugal

## Abstract

Porous hydrogels of poly(ethylene glycol) (PEG) have been shown to facilitate vascularized tissue formation. However, PEG hydrogels exhibit limited degradation under physiological conditions which hinders their ultimate applicability for tissue engineering therapies. Introduction of poly(_L_-lactic acid) (PLLA) chains into the PEG backbone results in copolymers that exhibit degradation via hydrolysis that can be controlled, in part, by the copolymer conditions. In this study, porous, PEG-PLLA hydrogels were generated by solvent casting/particulate leaching and photopolymerization. The influence of polymer conditions on hydrogel architecture, degradation and mechanical properties was investigated. Autofluorescence exhibited by the hydrogels allowed for three-dimensional, non-destructive monitoring of hydrogel structure under fully swelled conditions. The initial pore size depended on particulate size but not polymer concentration, while degradation time was dependent on polymer concentration. Compressive modulus was a function of polymer concentration and decreased as the hydrogels degraded. Interestingly, pore size did not vary during degradation contrary to what has been observed in other polymer systems. These results provide a technique for generating porous, degradable PEG-PLLA hydrogels and insight into how the degradation, structure, and mechanical properties depend on synthesis conditions.

## Introduction

Hydrogels have been investigated extensively for tissue engineering applications primarily due to mechanical properties of similar magnitude to many soft tissues [1], [2]. Poly (ethylene glycol) (PEG)-based hydrogels have received significant attention due to their biocompatibility, the relatively straightforward options for incorporation of peptide adhesion sequences [3] and growth factors, [4] and the ability to control mechanical properties based on polymerization conditions. While significant amounts of research have shown how modifications in the chemical composition of PEG hydrogels can modulate biological response, there has been little research into the role of the physical architecture.

Porous structure of biomaterials has been shown to play a role in regulating cell response and tissue integration. However, unlike ECM where cells can often migrate through pores between the solid structure, the cross-links in PEG hydrogels must be cleaved to enable cells to migrate and tissue to invade [1], [8]. Introduction of pores into PEG hydrogels could be used to improve biological response and lead to improved outcomes in biomedical applications. The introduction of pores not only provides more space for cell migration and tissue invasion but also increases the surface area to volume ratio which can enhance cell seeding [5] and enable more efficient mass transport. [6] A number of strategies have been employed to generate porous materials, including gas foaming [7], polymer-polymer immiscibility [8], and particulate leaching [9]. These techniques are most commonly applied to hydrophobic polymer scaffolds, but recent studies have demonstrated that hydrogels with an interconnected porous structure can be produced using particulate leaching techniques [10], [11]. PEG hydrogels generated with this technique support the formation of vascularized tissue *in vivo* in a pore size dependent manner [1]. While these materials have shown promise for tissue engineering, PEG hydrogels do not exhibit significant degradation *in vivo*.

PEG hydrogels can be made degradable under physiologic conditions through the inclusion of hydrolysable monomer units [12] or peptide sequences that are degraded by cell enzymes [12], [13]. While enzymatically degradable PEG hydrogels are popular as ECM mimics, they restrict cell migration and tissue invasion to enzymatic processes and can result in significant intrasubject variability. Materials degraded by hydrolysis allow for more controlled and less variable degradation kinetics. Poly (_L_-lactic acid) (PLLA) is a hydrophobic, biodegradable polymer [14], [15] that can be introduced into PEG systems to allow for controlled degradation via hydrolysis [16]. Hydrogels formed by polymerization of poly(ethylene glycol)-co-(_L_-lactic acid) diacrylate (PEG-PLLA-DA) degrade into products that are easily processed by the body [17]. Porous PEG-PLLA-DA hydrogels could maintain many of the advantages of porous PEG systems while exhibiting controlled degradation properties.

While various biodegradable porous scaffolds have been studied extensively [18], [19] these studies have largely focused on hydrophobic foams. There has been little evaluation of the structure of porous hydrogels and the influence of the degradation process on their properties. In addition, the majority of imaging techniques require destruction or modification of the samples from their native state in order to image. Autofluorescence exhibited by PEG-PLLA-DA hydrogels [20] allows the unique opportunity to monitor the 3D architecture of a porous hydrogel during the degradation process in fully swelled conditions.

Our goal is to optimize the design of porous hydrogels that coordinate the processes of vascularized tissue invasion with polymer degradation. In order to achieve this goal we must first gain an understanding into the properties of porous hydrogels and how they change during degradation. Here, we applied a particulate leaching technique to generate porous PEG-PLLA-DA hydrogels and examined the influence of polymer concentration and particulate size on the mechanical properties, pore structure, and degradation rate of the resultant hydrogels. To our knowledge, this is the first study that was able to evaluate the structure of porous hydrogels during degradation without sample processing or labeling with exogenous agents. This information could be used to help optimize hydrogel design for applications in tissue engineering.

## Experimental Section

### Materials

PEG (Mn ≈ 3400), stannous octoate, acryloyl chloride (98%), PKH26GL, 3,6-Dimethyl-1,4-dioxane-2,5-dione, triethylamine (99.5%), and 2-hydroxy-2-methylpropiophenone (Irgacure 1173) were obtained from Sigma (St. Louis, MO). Sodium chloride (99.5%), diethyl either, dichloromethane (DCM) anhydrous (99.9%), magnesium sulfate anhydrous (97%) and ethyl ether (anhydrous) were from Fisher Scientific (Pittsburgh, PA).

### Synthesis of PEG-PLLA-DA

The method to synthesize PEG-PLLA-DA was performed as described by Chiu *et al*. [21] Briefly, all glassware were dried in a vacuum oven at 120°C for 24 hours and cooled under vacuum. Ten grams of PEG (MW = 3400) and 2.12 g of 3,6-Dimethyl-1,4-dioxane-2,5-dione were placed into a 50 mL centrifuge tube and lyophilized. A round bottom flask was vacuumed and filled with argon. The lyophilized PEG and 3,6-Dimethyl-1,4-dioxane-2,5-dione were placed in the flask and then 80 µL of stannous octoate added as an initiator. The flask was submerged in a constant temperature oil bath at 140°C for 4 h. The product was then dissolved in 20 mL of anhydrous DCM and filtered using a GF/F filter. The resulting polymer was precipitated in 1.5 L of ice-cold diethyl ether three times and lyophilized. Based on ^1^HNMR analysis, these conditions result in approximately 10 lactide units per PEG macromer.

The lyophilized PEG-PLLA was acrylated as described previously. [21] Briefly, a three neck round bottom flask was vacuumed and filled with argon. Ten grams of PEG-PLLA was placed in the flask. Sixty mL of anhydrous DCM was injected into the flask, and 0.67 mL of triethylamine was added and stirred for 5 minutes. Acryloyl chloride (0.76 mL) was added dropwise. The flask was allowed to react for 24 hours at room temperature in the dark. The product was washed with 9.52 mL of 2 M K_2_SO_4_ and allowed to separate overnight. The organic phase was collected and precipitated in 2 L of ice-cold diethyl either. The extent of reaction, structure and purity of the products were determined by ^1^H NMR (Advance 300 Hz; Bruker, Billerica, MA). Products were dissolved in CDCl3 for ^1^HNMR with 0.05% v/v tetramethylsilane (TMS) used as an internal standard. Acrylation efficiency was 93±2%.

### Porous PEG-PLLA-DA Hydrogel Generation

The method for generating porous PEG-PLLA-DA hydrogels involved a salt leaching procedure with the polymer dissolved in an organic solvent. Lyophilized PEG-PLLA-DA polymer was dissolved in 1 mL of DCM and 2-hydroxy-2-methyl-propiophenone added as a photoinitiator (5% (w/v)). 250 mg of sieved salt and 250 µL of precursor were placed in a 1.5 mL centrifuge tube. The tube was vortexed for 45 seconds and placed upside down allowing the salt to settle in to the cap for 20 seconds. The concentration of PEG-PLLA-DA was varied from 12.5 to 50% (w/v) and the salt crystals used were selected by sieving in the following ranges: 150–100, 100–50 and 50–25 µm.

A microscope slide was used to cover the solution, carefully avoiding bubble formation. The solution was polymerized by irradiation under UV for 10 minutes. The sample was rotated 180° and polymerized for an additional 10 minutes. The microscope slide was removed and the DCM evaporated in a fume hood overnight. Resulting gels were placed in a 50 mL sterile centrifuge tube with 20 mL DI water with 4 mg/mL of gentamicin sulfate, and then immediately exposed to a vacuum (0.035 mBar) for 15 minutes to remove air trapped in the porous gels and to replace DCM with water. Water was changed 2 times a day until the salt was completely leached out.

### Swelling Tests

The porous PEG-PLLA-DA hydrogels were placed in individual 15 mL tubes with 5 mL of PBS (2% sodium azide and 4 mg/mL of gentamicin) and incubated at 37°C. PBS was changed every day until hydrogels completely degraded. Porous PEG-PLLA-DA hydrogels were weighed at various time points.

### Structural Analysis

The structure of porous PEG-PLLA-DA hydrogel could be imaged by confocal microscopy due to autofluorescence exhibited by the hydrogels [20]. A PASCAL laser scanning microscopy system from Carl Zeiss MicroImaging, Inc. (Thornwood, NY), was used for confocal imaging. The hydrogel was imaged using a 488 nm laser with a 505 nm low pass filter. Images had *x* and *y* resolution of 3.5 µm/pixel and *z* resolution of 1.8 µm/pixel. The samples were scanned 180 µm deep from the surface at 10 µm intervals, collecting 18 slices in total. Each stack was imported into AxioVision 4.5 (Carl Zeiss, Göttingen, Germany) in order to allow quantification of pore size. Pore size was defined as the longest axis of a given pore and was selected with the built-in caliper tool in AxioVision. Ten pores were selected at each 10 µm thick slice and pore size pooled with the values obtained from the other 19 slices. The average of the pooled values is the pore size value for that sample at that time point. This process is repeated for all samples at each time point.

### Compression Testing

Compression testing was conducted at a constant strain rate of 0.5 mm/min using a RSA3 (TA Instruments) [9], [22]. Samples were formed to match the plate size (15 mm) and then compressed. The strain and normal force were recorded and used to calculate the compressive modulus for each sample. The initial diameter and area of each gel were measured and recorded before testing. Compressive moduli of the gels were found by plotting a stress-strain graph with the strain going up to 0.1 or 10%. Strain zero corresponds to the first acceptable stress value. None of the gels were fractured during compression.

### RGD Conjugation

The method for conjugation of peptides to acryl-PEG was performed as described previously [21]. A solution of 50 mM NaHCO_3_ (pH 8.3) was prepared as a buffer. Ten milligrams of YRGDS (American Peptide, Sunnyvale, CA) was dissolved in 5 mL of 50 mM NaHCO_3_. Acryl-PEG-SVA (3400 Da; Laysan, Arab, AL) was dissolved in 7 mL of 50 mM NaHCO_3_ and then added drop-wise into the stirred YRGDS solution in the dark. The molar ratio of YRGDS to acryl-PEG-SVA was 1∶1.5. The solution was stirred for 2 h at 4°C in the absence of light. The final product was dialyzed (2000 Da molecular weight cut-off) in 2 L of DI water for 24 h (with replacement after 12 h). The resulting product was lyophilized and stored at −80°C until use.

### Cell Culture

The cell culture and cell seeding methods have been described previously. [10] Briefly, NIH 3T3 fibroblasts (Cambrex, Walkersvile, MD) were maintained in complete media (Dulbecco’s modified Eagle’s medium, 10% fetal bovine serum, and 1% penicillin–streptomycin). The cells were passed when flasks reached 90% confluency. Gels were placed into 48-well plates, and incubated in complete media for 1 h. After removing media, gels were air dried in a culture hood for 1 h. Five thousand PKH26 stained 3T3 fibroblasts in 0.5 ml was added directly to the gel surface. Samples were incubated at 37°C, 5% CO_2_ overnight and imagined at varying time points. Gels were imaged using confocal microscopy (488 nm laser with a 505 nm low pass filter).

### Statistics

Data are presented as means ± standard deviation. Significant differences between groups of data were determined by analysis of variance with Holm-Sidak post-test. In all cases, *p*<0.05 was considered statistically significant.

## Results

### The Influence of Polymer Concentration on Polymer Properties

**Figure 1:**
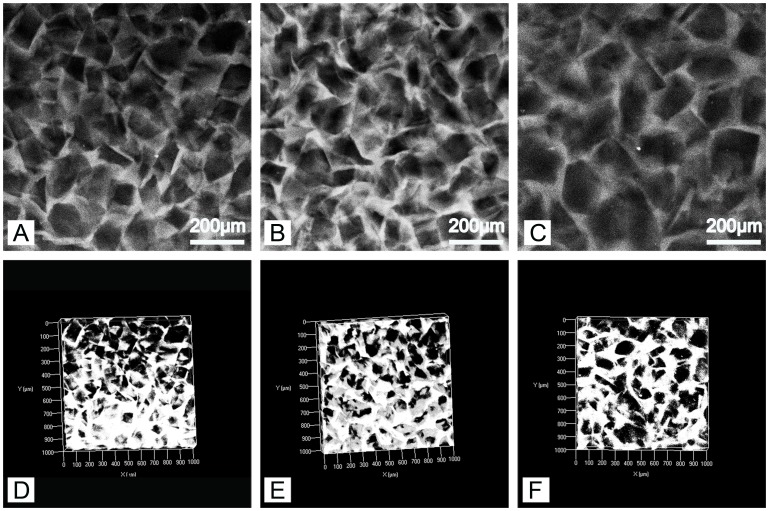
Two dimensional slices (A–C) and volume renderings (D–F) of 12.5% (w/v) porous PEG-PLLA-DA hydrogels generated with salt crystal size ranging from 150–100 µm at 1 (A,D), 3 (B,E), and 7 (C,F) days of incubation.

**Figure 2:**
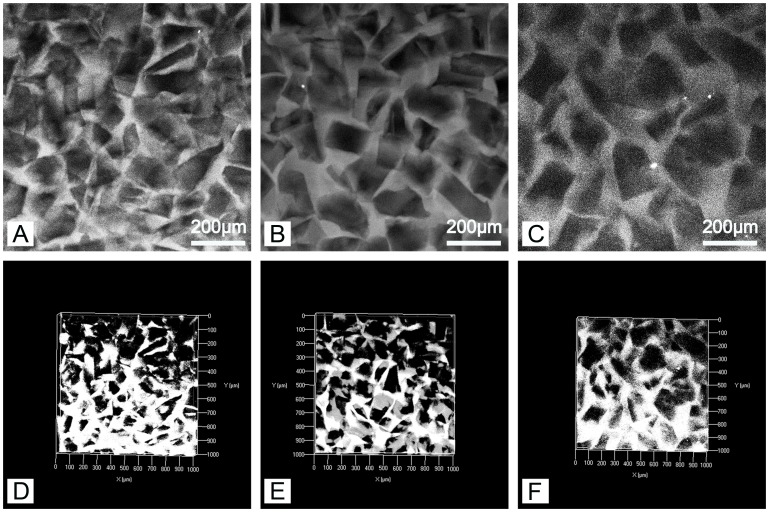
Two dimensional slices (A–C) and volume renderings (D–F) of 25% (w/v) porous PEG-PLLA-DA hydrogels generated with salt crystal size ranging from 150–100 µm at 1 (A,D), 7 (B,E), and 14 (C,F) days of incubation.

**Figure 3:**
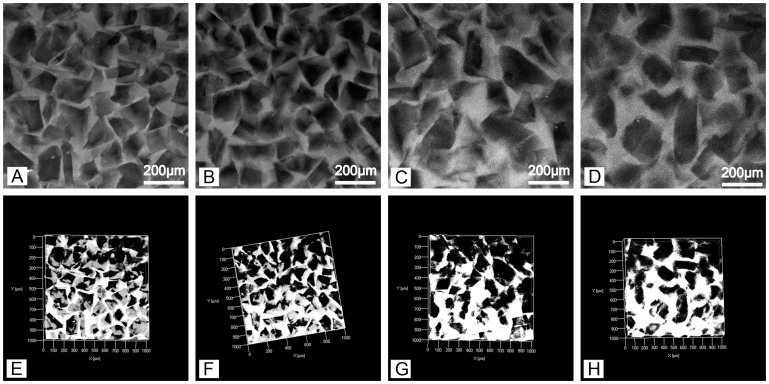
Two dimensional slices (A–D) and volume renderings (E–H) of 50% (w/v) porous PEG-PLLA-DA hydrogels generated with salt crystal size ranging from 150–100 µm at 1(A,E), 7(B,F), 14(C,G), and 21(D,H) days of incubation.

The autofluorescence of PEG-PLLA-DA allows imaging of hydrogel structure nondestructively using confocal microscopy [20]. This is highly advantageous relative to traditional techniques for characterizing material structure, such as scanning electron microscope (SEM), because it avoids processing of samples by drying or fixation that may alter the architecture. We first investigated the effect of polymer concentration on hydrogel properties. Samples were generated with the same range of particulate size (150–100 µm) at varying polymer concentrations of 12.5, 25, and 50% PEG-PLLA-DA. Figure 1 displays confocal images of 12.5% (w/v) porous PEG-PLLA-DA hydrogels at days 1, 3, and 7. The structure of porous hydrogels at different time points can also be seen for 25% (Fig. 2) and 50% hydrogels (Fig. 3).

**Figure 4:**
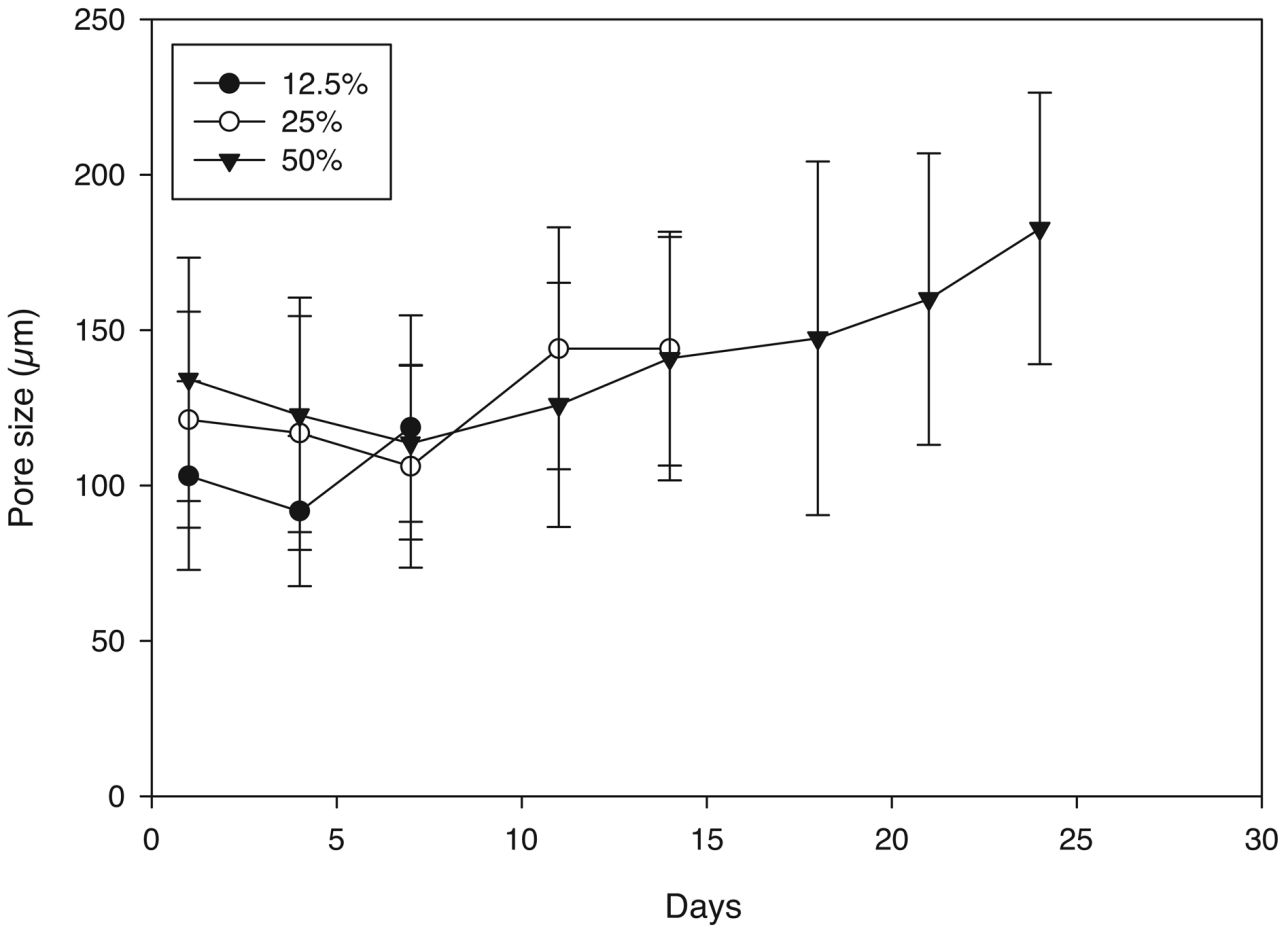
Mean pore size of porous PEG-PLLA-DA hydrogel generated with salt size ranging from 150–100 µm at various PEG-PLLA-DA percentages plotted versus incubation time in vitro.

The confocal images show a porous structure in all hydrogels throughout the degradation process. At the first time point, the hydrogels exhibited pores with structure and size consistent with the salt crystals used as the pore agent. Interestingly, the intensity of the autofluorescence decreased as the hydrogels degraded. For example, at early time points (Fig. 2 A&D), confocal images were bright and hydrogel structures could be easily discerned. By day 14, the intensity of the images decreased and borders appeared blurry (Fig. 2 C&F). Regarding polymer concentration, the 50% group (Fig. 3 B) had higher autofluorescence compared to the 25% group (Fig. 2 C) likely due to higher polymer concentration. However, even with the lower fluorescence signal from the later time points, quantification and analysis of scaffold architecture was still possible from the confocal images (Fig. 4). The size of the pores remained constant throughout the majority of the degradation time for all conditions. Prior to complete degradation of the hydrogels there was a slight increase in mean pore size.

**Figure 5:**
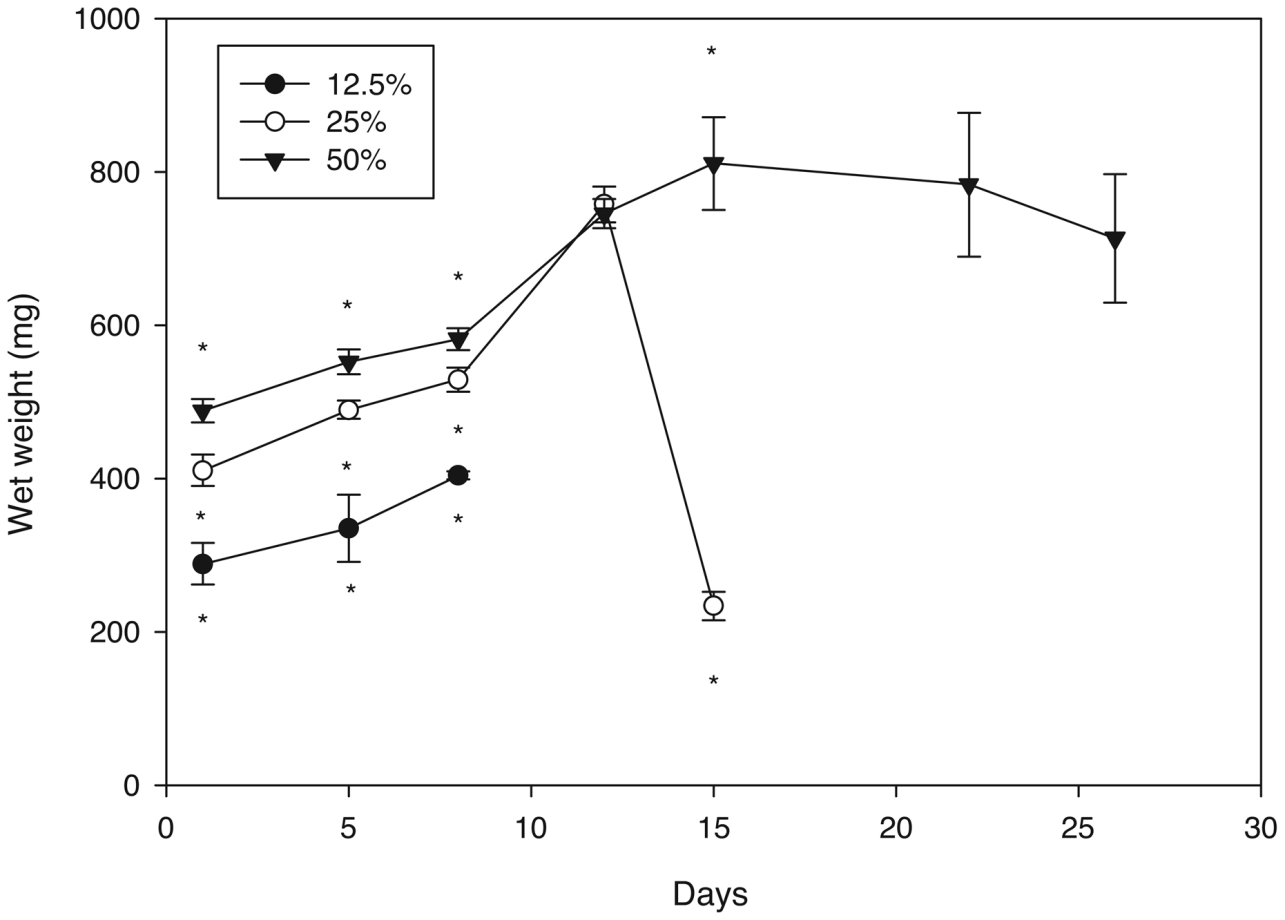
Wet weight of porous PEG-PLLA-DA hydrogels versus time for hydrogels generated with salt size ranging from 150–100 µm at various polymer concentrations (*indicates statistical difference between all groups at that time point, p<0.001). The significant reduction in weight seen on day 15 for 25% gels is due to the fact that these gels were highly degraded at that time point, and the gels had reduced greatly in size.

The wet weight of the hydrogels was also quantified which provides information on the degradation rate (Fig. 5). The initial wet weight depended on the percentage of polymer used and increased as the materials degraded. The time to complete degradation varied with polymer concentration, with 12.5, 25, and 50% gels degraded in 7, 16, and 26 days respectively.

### The Influence of Polymer Concentration on Mechanical Properties

**Figure 6:**
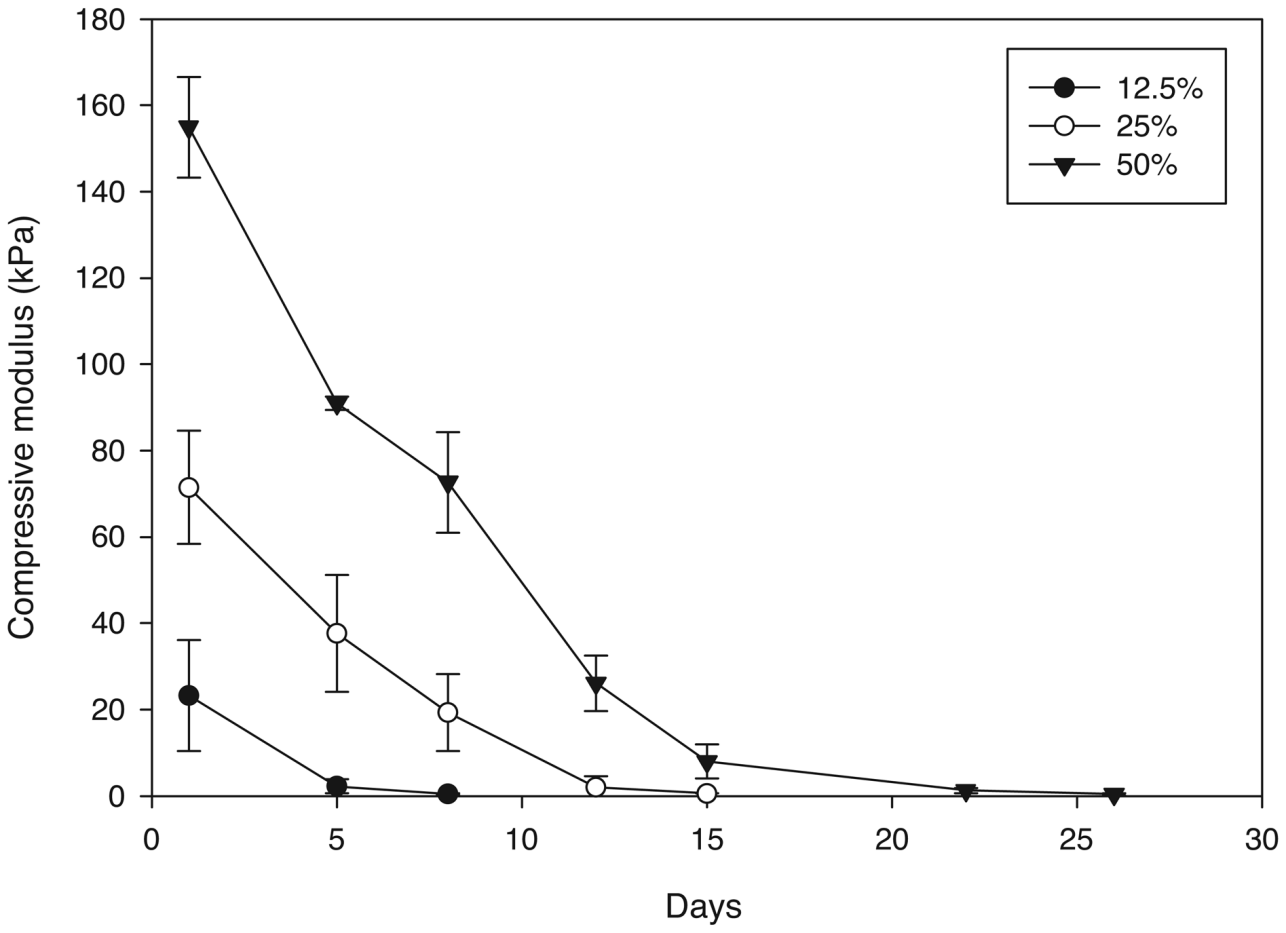
Sample stress-strain curves of 25% (w/v) porous PEG-PLLA-DA hydrogels generated with salt sizes ranging from 150–100 µm at days 1, 3, 5, 8,and 10.

**Figure 7:**
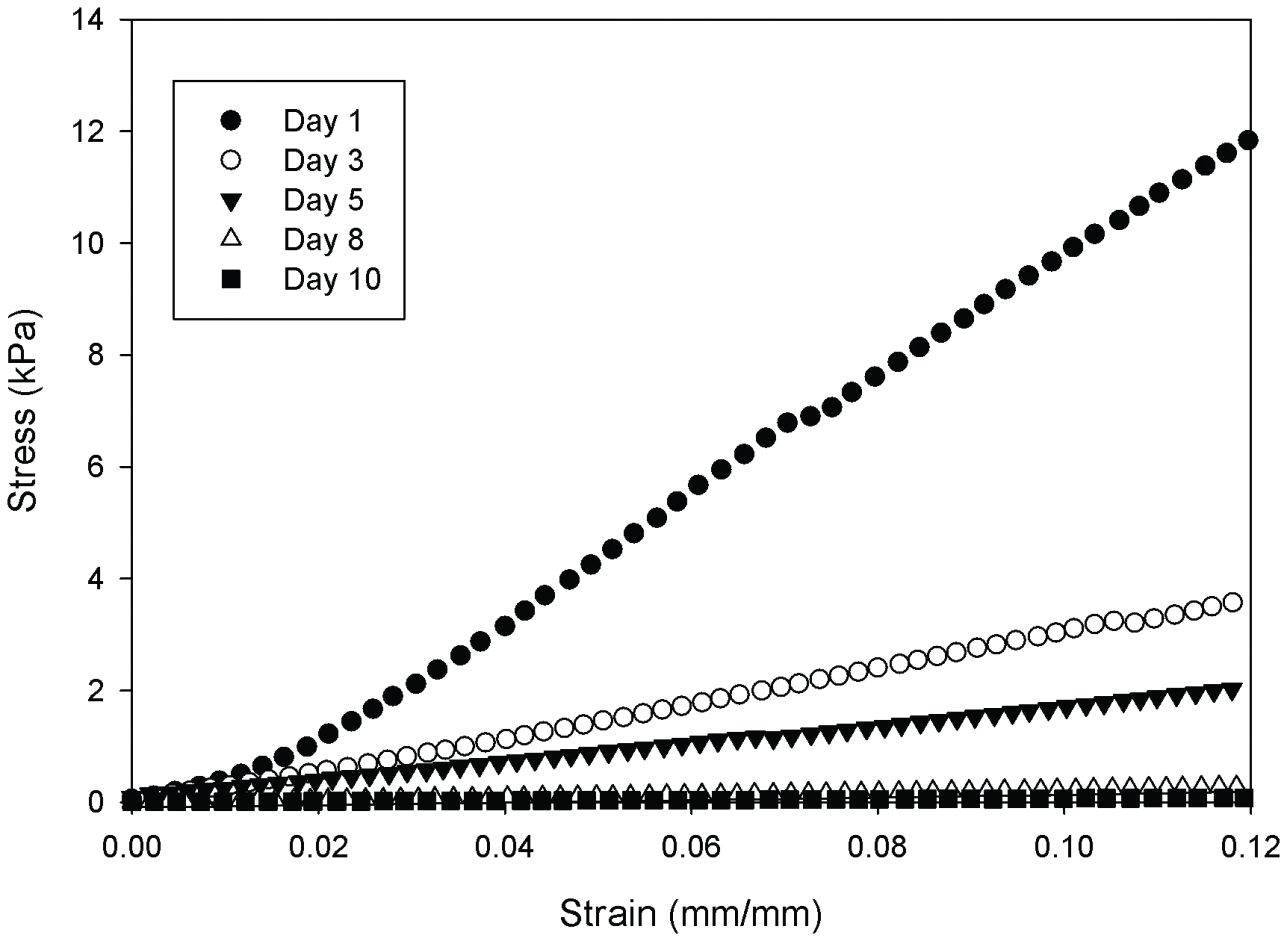
Compressive moduli of porous PEG-PLLA-DA hydrogels generated with salt sizes ranging from 150–100 µm with varying PEG-PLLA-DA percentages plotted versus time. Compressive moduli are statistically different points between all groups at each time point (p<0.001).

The compressive moduli of 12.5, 25, and 50% porous PEG-PLLA-DA hydrogels, generated with salt size ranging from 150–100 µm, were quantified. Figure 6 shows typical curves generated for the hydrogels, illustrating the rapid decrease in stiffness as the hydrogels degrade. At day 1 there were significant differences in mechanical properties between 12.5, 25, and 50% porous PEG-PLLA-DA hydrogel at the same pore size (150–100 µm) (Fig. 7). The compressive modulus was higher for hydrogels with greater polymer content. The compressive moduli decreased rapidly as the hydrogels degraded (Fig. 7).

### The Influence of Particulate Size on Pore Structure and Degradation

**Figure 8:**
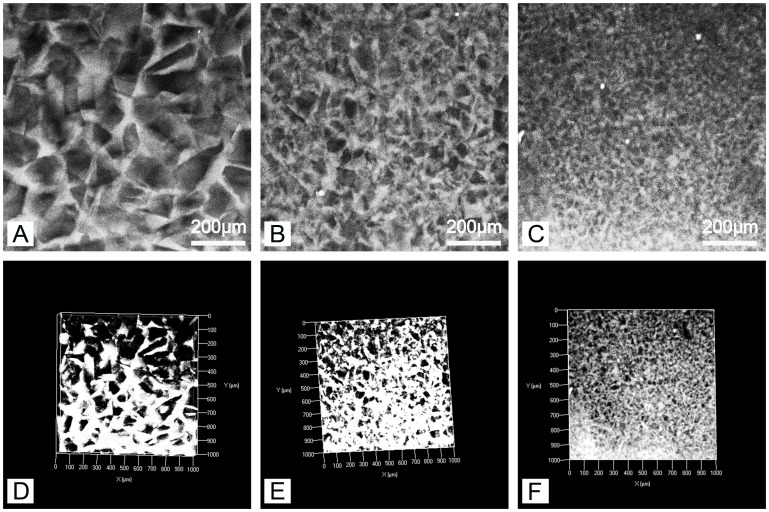
Two dimensional confocal sections (A–C) and three dimensional volume renderings (D–F) of porous hydrogels. The 25% (w/v) hydrogels were generated with salt crystal sizes ranging from150–100 µm (A,D), 100–50 µm (B,E), and 50–25 µm (C,F) of incubation.

We also investigated the effect of pore size on the mechanical and degradation properties of the hydrogels. Twenty-five percent PEG-PLLA-DA hydrogels were generated with salt crystal sizes ranging from 50–25 µm, 100–50 µm, and 150–100 µm. The structure of the hydrogels imaged under swelled condition can be seen in Figure 8. In all cases, an interconnected porous structure is apparent with pore size increasing with the salt crystals used. The size and shape of the pores within the hydrogels were consistent with the salt crystals used as the pore forming agent.

**Figure 9:**
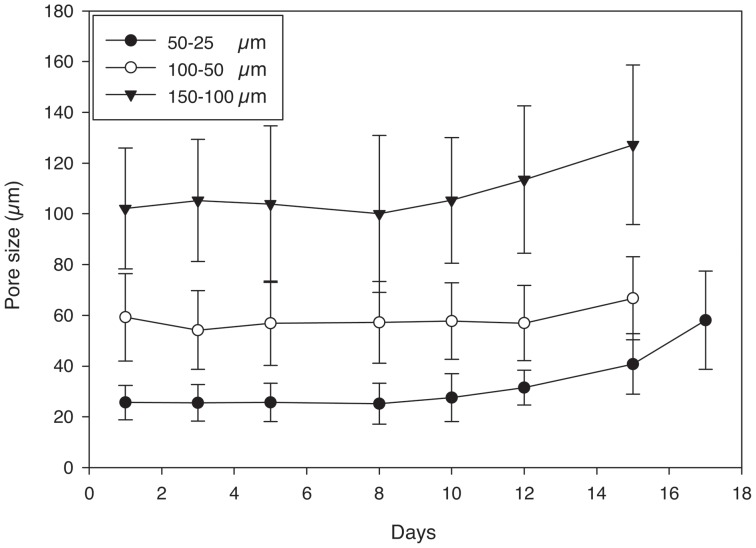
Mean pore size versus time for 25% (w/v) porous PEG-PLLA-DA hydrogels generated with salt crystal sizes ranging from 150–100 µm, 100–50 µm, and 50–25 µm.

**Figure 10:**
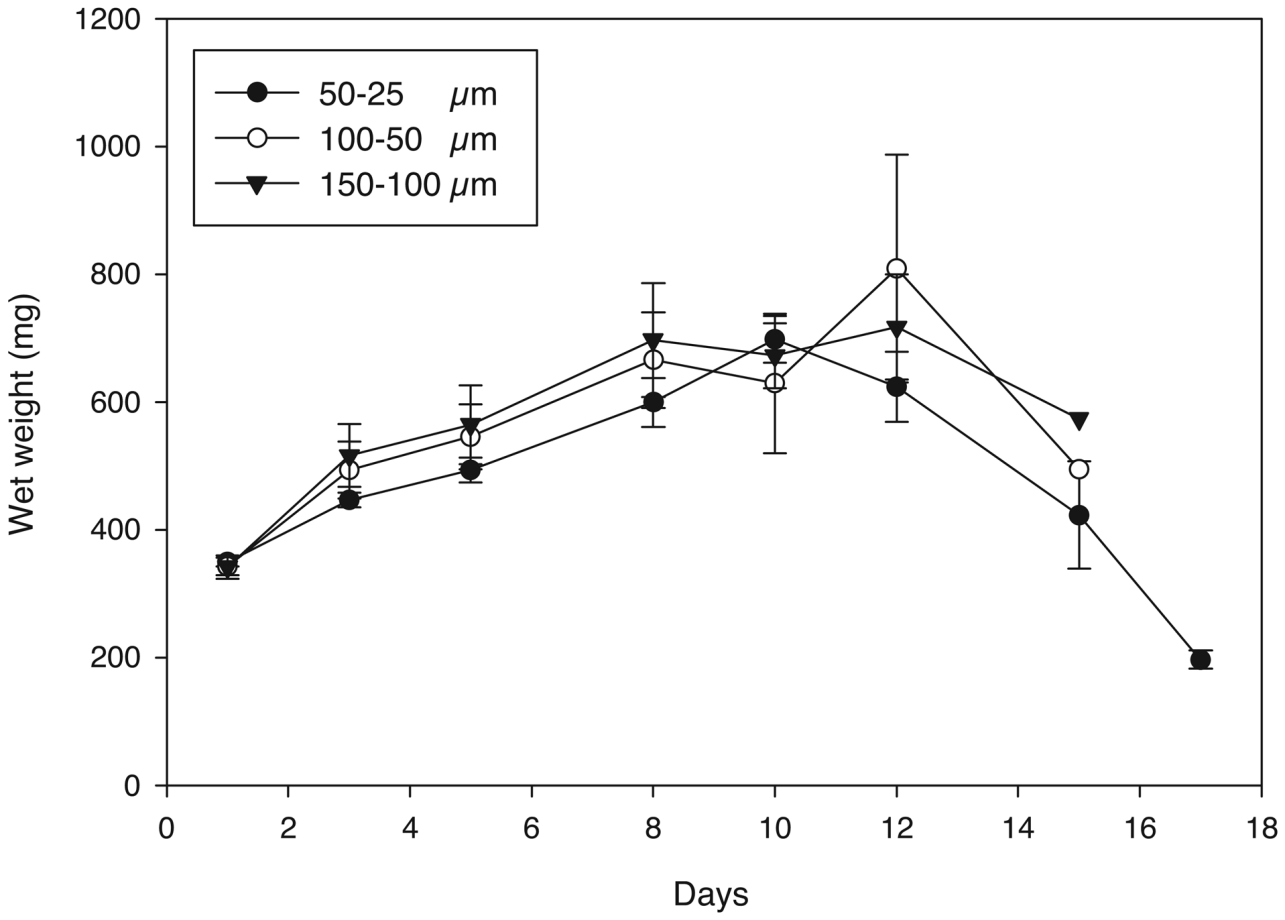
Wet weight versus incubation time for 25% (w/v) porous PEG-PLLA-DA hydrogel generated with varying salt sizes.

Pore sizes agreed well with salt crystal sizes at day 1 (Fig. 9) and the mean pore size remained constant for most of the degradation process with a slight increase prior to complete hydrogel degradation (Fig. 9). The wet weight initially increased as the hydrogels degraded and then decreased towards the end of the process. The degradation rate of the hydrogels did not appear to depend on pore size as all conditions degraded in 15–17 days (Fig. 10).

### The Influence of Particulate Size on Mechanical Properties

**Figure 11:**
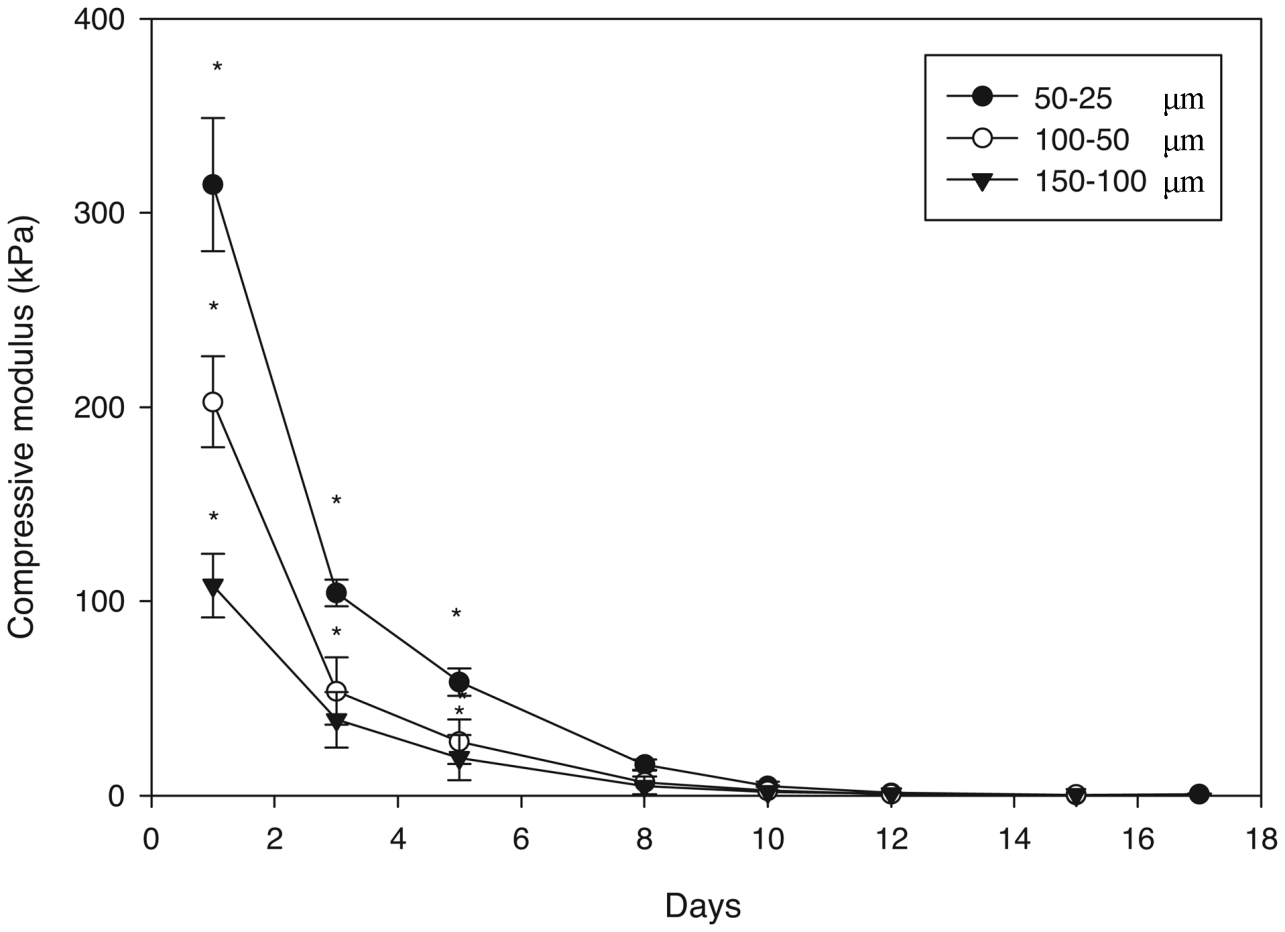
Compressive moduli of 25% (w/v) porous PEG-PLLA-DA hydrogels generated with varying salt sizes during in vitro degradation (*indicates statistical differences between groups) (p<0.001).

The stiffness of the gels decreased throughout degradation (Fig. 11). The compressive moduli were significantly different between the different pore sizes at time points up to one week (*p*<0.001). Gels with smaller pores were stiffer then gels with larger pores, and the stiffness of the gels decreased rapidly throughout degradation.

### The Influence of Copolymer Concentration on Cell Adhesion

**Figure 12:**
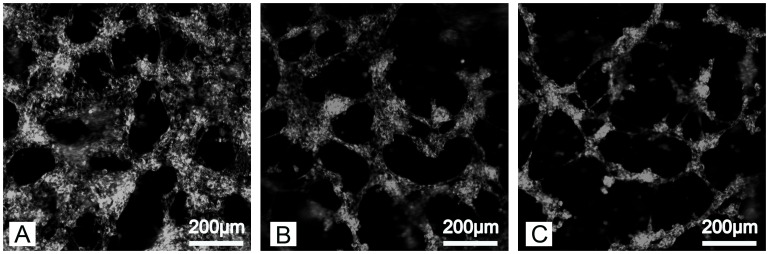
Confocal images of PKH 26 stained fibroblasts adhering to porous hydrogels. The hydrogels were generated using salt crystals ranging in size 150–100 µm but polymer concentrations of A) 50%, B) 25%, and C) 12.5%.

**Figure 13:**
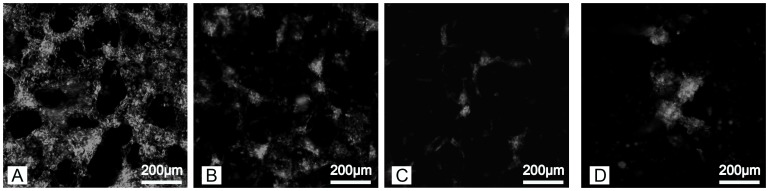
Confocal images of PKH 26 stained 3t3 fibroblasts on 50% porous hydrogels made with crystal size 150–100 µm at days A) 1, B) 5, C) 10, and D) 22 of incubation.

A cell adhesion sequence (YRGDS) was incorporated in the hydrogels to determine whether the porous gels could support the adhesion of cells. Fibroblasts spread and lined the edges of pores in all polymer conditions (Fig. 12). Cells on 50% porous hydrogels made with crystal size 150–100 µm were imaged over time (Fig. 13). At day 1, cells appeared to line the edge of the pores (Fig. 13A). The cell organization changed as the gels degraded and, by day 22, cells had formed multicellular aggregates within the gels prior to complete degradation (Fig. 13 C).

## Discussion

The ability to modulate and control tissue response to implanted biomaterials is essential to the fields of tissue engineering and regenerative medicine. We have previously investigated porous PEG hydrogels and found that these hydrogels support vascularized tissue formation [1]. Under the conditions investigated, hydrogels with pores ranging from 150–100 µm supported the most rapid vascularization *in vitro* and *in vivo.* In these studies, there were no signs of degradation exhibited by either porous or non-porous PEG hydrogels. However, the success of these materials in clinical applications requires that they degrade in a controlled fashion as new vascularized tissue develops.

Hydrogels generated from PEG-PLLA-DA copolymers have been investigated in many applications in regenerative medicine. Materials based on PEG-PLLA copolymers have been applied as biological coatings, tissue engineering scaffolds, and drug delivery systems, but there has been little investigation into the design and optimization of PEG-PLLA-DA hydrogels with porous structure [23]. The polymer conditions used here are similar to those that have been described in other studies [24]. However, the generation of pores offers a number of advantages over nonporous structures. This includes enhanced nutrient transport and higher surface area to volume ratio. While these hydrogels do not allow invasion via protease-mediated degradation [25], the reliance on hydrolytic degradation allows the potential to decouple tissue invasion from hydrogel degradation. In this study, we generated porous PEG-PLLA-DA hydrogels by solvent casting with DCM, particulate leaching, and photopolymerization. This particulate leaching technique has been commonly used for hydrophobic polymer foams, but we show that it also serves as a simple method for generating pores in PEG-PLLA-DA hydrogels.

Studies have been performed examining the structure of hydrophobic polymer foams during degradation, but research has not been performed into porous hydrogels systems. The equal availability of water throughout the polymer volume results in differences in structural changes as the materials degrade relative to hydrophobic materials. Autofluorescence exhibited by the PEG-PLLA-DA allowed the unique ability to characterize 3D polymer structures when they are fully swelled which are the conditions used in bioreactors and cell culture [10], [20]. The origin of this autofluorescence is still not clear but it appears to result from a synergetic effect of both lactate units and diacrylate groups in the PEG-PLLA-DA backbone. However, the fluorescence not only allows imaging of the polymer structure with confocal microscopy but can be exploited to monitor degradation as the intensity is proportional to the number of PEG-PLLA chains present. [20].

The hydrogels exhibited an interconnected porous structure with initial pore size correlating well with the size of the particulates selected. The pore size and structure remained consistent throughout degradation and did not depend on polymer concentration. In studies with hydrophobic polymer foams, pores have been shown to decrease in size and number while the scaffolds degraded [26]. In addition, the overall architecture of the pores in PLGA scaffolds changes, losing the crystal shape that results initially from the particle leaching technique. The hydrogels used in this study, however, maintained their size and structure as they degraded. While the dissolution of porous polymer foams exhibit a bulk degradation mechanism, the change in pore structure occurs as the chains on the surface of the pore having greater access to water than those within the structure. This results in more rapid degradation at the surface of the pore and a change in size throughout degradation. Consistent with experimental and computational models of nonporous PEG-PLLA hydrogels [27], [28], the porous hydrogels exhibit a bulk mechanism of degradation which means that the overall structure, including pore size, is maintained up to the point of a nearly instantaneous dissolution of the final volume. The rapid decrease observed in wet weight in the 25% hydrogel is an indication of this rapid dissolution. However unlike the polymer foams, hydrogels rapidly absorb water resulting in all chains having equal access to water whether they are on the surface of a pore or part of the bulk structure. Swelling ratio studies support the concept that pore size does not influence access to water. Hydrogels swell as they degrade [29] and the wet mass was independent of pore size at all time points. The equal access of the polymer chains to water allows maintenance of pore structure as the hydrogels degrade until they reach the point of complete dissolution.

The compressive moduli of the hydrogels depended on both pore size and polymer content. As expected, the modulus increased with polymer content, which agrees with literature showing increasing crosslink density with polymer content [30]. In addition, the stiffness decreased with increasing pore size. This result agrees with previous studies which investigated the influence of pore size on porous poly (propylene fumarate) scaffolds [9]. Increasing polymer concentration could increase the mechanical properties of the hydrogels while keeping pore size constant. The mechanical properties also diminished rapidly during incubation suggesting a bulk mechanism of degradation, which is consistent with swelling and pore size observations. We have previously shown that pore size plays an important role in biological response to porous PEG-based hydrogels [1]. However, mechanical properties and degradation time also contribute to biological response [31], [32]. While our results suggest varying polymer concentration may allow for the design of hydrogels with mechanical properties independent of pore size, it does not appear the degradation time can be easily decoupled from mechanical properties using this approach.

Porous hydrogels supported cell attachment under all conditions following the incorporation of cell adhesion sequences. As the hydrogels degraded the structure of the cells in the gels changed. The cells eventually assembled into aggregates before the complete degradation of hydrogels. The change in cell morphology is somewhat surprising considering that the pore size and structure do not change as the gels degraded. However, the reduction in hydrogel stiffness and possible local change in ligand density with degradation could influence cell behavior. It is well-established that cell migration is influenced by hydrogel stiffness [33] and cells exhibit a round shape and less stress-fiber formation in polyacrylamine gels with lower stiffness [34]. Cell behavior also is dependent on ligand density [35], which may change locally as degradation reduces the dangling ligands present in the gels [36]. These results suggest how changes in hydrogel properties despite constant pore structure could change biological response to the materials. Future studies will examine cell behavior and tissue formation within porous PEG-PLLA-DA hydrogels and the role of stiffness and ligand density on the response.

### Conclusion

A technique was developed for the application of salt leaching methods to generate porous PEG-PLLA-DA hydrogels. This study demonstrates the influence of polymer concentration and pore size on the mechanical, structural, and degradation properties of these porous hydrogels. The architecture of porous PEG-PLLA-DA hydrogels was monitored without drying or destruction by using the polymer’s intrinsic fluorescence. Interestingly, this allowed for the determination that pore size and structure remained constant during degradation. Results from this study provide a better understanding of the mechanical property and architecture of porous PEG-PLLA-DA hydrogels during degradation, which helps to design biodegradable, porous scaffolds for tissue engineering.
